# Female circumcision: Limiting the harm

**DOI:** 10.12688/f1000research.1-23.v2

**Published:** 2012-11-08

**Authors:** Mohamed Kandil

**Affiliations:** 1 The Department of Obstetrics and Gynecology, Faculty of Medicine-Menofyia University, Shibin Elkom, Egypt

## Abstract

**Objective:** To review the strength of evidence that links many health hazards to female genital cutting.

**Material and methods:** Literature search in Medline/Pubmed and Google scholar.

**Results:** Female genital cutting is still practiced secretly in both underdeveloped and developed countries due to prevailing strong traditional beliefs. There is insufficient evidence to support the claims that genital cutting is a harmful procedure if performed by experienced personnel in a suitable theatre with facilities for pain control and anesthesia. Cutting, however, is advised not to go beyond type I.

**Conclusion:** Law makers around the globe are invited to review the legal situation in relation to female genital cutting. Proper counseling of parents about possible risks is a must in order to make informed decision about circumcising their daughters. The procedure should be offered to parents who insist on it; otherwise, they will do it illegally, exposing their daughters to possible complications.

## Introduction

Female genital cutting/mutilation (FGC/M), or circumcision as it was previously described ^1^, is held responsible for a multitude of health risks. According to WHO, FGC/M is defined as “all procedures that involve partial or total removal of the external female genitalia, or other injury to the female genital organs for non-medical reasons” ^2^.

The legislations enacted in most countries to ban FGC had minimal effect on its prevalence ^3^. In the most recent estimate carried out by the WHO in 2008, an average of between 100 and 140 million women have undergone FGC in the world and every year, 3 million female children are mutilated in Africa ^4^.

## Female genital cutting in medical literature

I searched the English literature in Medline/Pubmed and Google Scholar for female genital cutting/mutilation and circumcision in the period from January 1980 until January 2012. The available studies showed that FGC may result in either physical and/or psychological injuries, immediate and/or late.

## Alleged health hazards

### Immediate complications

The three immediate complications are bleeding, pain and infection. They are not unique to FGC. They are liable to occur with any other type of female surgery, whether minor or major. Bleeding is liable to occur with the tiniest injury to the body, not only genitalia, and death may occur if not dealt with. Pain during genital cutting was attributed to non use of anesthesia or pain killers during the procedure ^5^, something which is expected with any other similar situation. The procedure is illegal in most countries of the world and it is routinely performed at home using non-sterilized instruments. Infection is the normal sequel for any surgical interference performed in such an environment. We should ask ourselves what would be the percentages of these complications if FGC was performed in a well-equipped theatre by experienced personnel. They would probably not be different to any other surgical procedure.

### Late complications

The alleged late risks include a wide variety of complications. Scars and keloid formation may occur ^6^. It is well known that the type of scar depends on the mode of healing, whether by primary or secondary intention. Healing with secondary intention and the formation of ugly scars occurs if the wound is left to heal on its own without repair. This pattern of healing is expected because the procedure is usually performed by the traditional illiterate birth attendant (IBA) at home. Epidermoid cysts may form probably due to cutting with non sharp instruments or imprecise cutting by the traditional IBA or un-experienced surgeon ^7^. The occurrence of both complications can be minimized if the procedure is performed in a well-prepared theatre. Controversy exists as for sexual pleasure. Although many researchers reported that female genital mutilation interferes negatively with women’s sexual pleasure, others provided contradictory evidence and confirmed that women with types I and II cuttings were able to enjoy their sex lives ^8, 9^. Lightfoot-Klein ^10^ conducted a study on infibulated females “type III cutting” in Sudan and, based on her findings, she stated that nearly 90% of all women said that they experienced orgasm or had experienced it at various periods in their marriage. Thabet et al. showed that women with type II cutting complain of defective sexuality compared to non circumcised women, while women with the more extensive type III cutting are not different to controls ^11^. This is not logical. If FGC is responsible for defective sexuality, those with type III cutting should have the maximum suffering. The explanation for this contradiction is because sexual arousal is not only dependent upon clitoral stimulation. It involves the stimulation of nerve endings in and around the vagina, vulva, cervix, uterus and clitoris, with psychological response and mindset also playing a role ^12, 13^.

There are claims that women who have undergone genital cutting may have a feeling of inferiority ^14^. This is apparent when these women immigrate to western societies which do not practice FGC. This psychological burden probably stems from the fact that their new societies consider FGC as abnormal contradicting the traditions and beliefs they have grown up with. There are other claims that infertility may also complicate FGC. Reasons are anatomic disfigurement due to excessive scarring after infibulation “type III” probably resulting from healing by secondary intention. Another cause is the associated infection; that might arise after FGC, to the internal genitalia causing inflammation and scarring and subsequent tubal block ^15^. Infection again is due to the improper environment where the procedure was performed.

The WHO reported that obstetric complications are more likely to occur with genital cuttings and the risk increases with more advanced cutting ^16^. This conclusion was based on a WHO collaborative prospective study which included 28,393 women attending for singleton delivery at 28 obstetric centers in Burkina Faso, Ghana, Kenya, Nigeria, Senegal, and Sudan. The WHO study and few others also showed that a higher percentage of cut women deliver by Cesarean section compared to uncut women due to an increased number of obstructed labors. There is a higher incidence of infant resuscitation, stillbirth, or neonatal death in mothers with FGC ^16– 18^. One of the major drawbacks of the WHO study is that the population studied is not representative for the whole population in the selected countries. In poor societies, only high-risk and complicated pregnancies are referred to hospitals. Such cases are more liable for adverse obstetric outcomes. This may have overestimated the rate of complications in women with FGC who attended hospitals to deliver. Claims for increased Cesarean deliveries in cut women were attributed to obstructed labor most likely due to excessive scarring at the pelvic outlet probably resulting from the imperfect healing of the genital cutting and possible associated infection. However, the high Cesarean rate in this population cannot be attributed solely to obstruction due to excessive outlet scarring; obstructed labor may occur due to a variety of reasons. In fact, excessive scarring at the pelvic outlet is the easiest reason to deal with, using a generous episiotomy. The reason for increased stillbirth and/or neonatal death in mothers with FGC is probably related to the obstructed labor; whatever the reason is, it is not a direct complication of FGC.

## Comments

The decline in FGC practice is not proportionate to the efforts exerted ^3^. It is not easy to give up your traditions and cultural beliefs for what is considered, by many, to be an attempt to westernize societies in the third world. Many believe that national and international feminist organizations and child rights’ advocates have propagated misleading or unproven information through the media in order to force governments to prohibit the procedure. In fact, all the above-mentioned health hazards were concluded from studies that showed inconsistent findings. Some of them confirmed the hazards of FGC while others failed to prove them. In the era of evidence based medicine, level I evidence, derived from either systematic reviews or randomized controlled trials (RCTs), to support the ban against FGC is not available. Such studies were never considered by the WHO or any other international health organization before the ban of FGM takes place in most countries. In fact, the design and implementation of a RCT to address the effects of FGC cannot be justified and seems to be unethical. In light of this fact and in the absence of any scientific evidence to support the practice of female circumcision, the available level III evidence, derived from retrospective studies and studies depended on self-reported FGC and its health consequences, should be taken into consideration in spite of their imprecision and low reliability ^19, 20^.

## Religious and cultural views

In Islam and Judaism, male circumcision is a must while female is not. In Islam, if female circumcision is desired by parents, it should not go beyond type I FGC (Ia is removal of the prepuce and Ib is removal of the prepuce and clitoris) according to hadith “Sunna type of circumcision”. This type of female genital surgery is equated with male genital surgery ^21^. In support of hadith, many studies showed that women with clitoridectomy “type I cutting” are less likely to develop gynecologic or obstetric complications compared to infibulated women “type III” ^6^. Considering that the number of Moslems in the world ranks second, it seems logical to reconsider the legal attitude towards female circumcision and probably avoids the ban directed towards Sunna circumcision.

It therefore seems that the prohibition of FGC for those who strongly believe in circumcision in the absence of solid scientific evidence does not respect their traditions and cultural beliefs. Women in societies which practice FGC and the practicing immigrant minorities living in the west consider that strength and identity partly come from the pain and difficulty which FGC causes, making them ‘strong’ and ‘desirable’ women ^22, 23^.

## Conclusions

To conclude, law makers all around the globe are invited to review the legal situation of female circumcision. Parents, especially immigrants to the western world from the practicing societies, should be properly counselled for the possible complications, but should also be informed that these data were not derived from randomized controlled trials. Those who insist on circumcising their daughters should be allowed to do so, but advised not to exceed type I cutting; otherwise, they will go for it secretly and illegally by inexperienced personnel in a poorly hygienic environment with the possibility of complications.

## References

[1] Rahman A Toubia N: Female Genital Mutilation: A practical Guide to worldwide Laws and Policies Worldwide.Published by Zed Books2000 Reference Source

[2] Female genital mutilation.World Health Organization. Fact sheet No 241, Geneva. February 2010. Last accessed 9 December 2011. Reference Source

[3] Cottingham J Kismodi E: Protecting girls and women from harmful practices affecting their health: Are we making progress? *Int J Gynecol Obstet.* 2009;106(2):128–31 10.1016/j.ijgo.2009.03.024 19535073

[4] Eliminating Female genital mutilation – An interagency statement (OHCHR, UNAIDS, UNDP, UNECA, UNESCO, UNFPA, UNHCR, UNICEF, UNIFEM, WHO), World Health Organization,2008; pp.4,22–28 Reference Source

[5] Female genital mutilation.World Health Organization(2008) Last accessed date 27 November 2011. Reference Source

[6] Kelly E Hillard PJ: Female genital mutilation. *Curr Opin Obstet Gynecol.* 2005;17(5):490–4 16141763 10.1097/01.gco.0000183528.18728.57

[7] Kroll GL Miller L: Vulvar epithelial inclusion cyst as a late complication of childhood female traditional genital surgery. *Am J Obstet Gynecol.* 2000;183(2):509–10 10.1067/mob.2000.105963 10942500

[8] Catania L Abdulcadir O Puppo V: Pleasure and Orgasm in Women with Female Genital Mutilation/Cutting (FGM/C). *J Sex Med.* 2007;4(6):1666–78 10.1111/j.1743-6109.2007.00620.x 17970975

[9] Nussbaum MC: Judging Other Cultures: The Case of Genital Mutilation. *Sex and Soc Justice.*Oxford University Press1999;pp.119–20 Reference Source

[10] Lightfoot-Klein H: The Sexual Experience and Marital Adjustment of Genitally Circumcised and Infibulated Females in the Sudan. *J Sex Res.* 1989;26(3):375–392 10.1080/00224498909551521

[11] Thabet SM Thabet AS: Defective sexuality and female circumcision: the cause and the possible management. *J Obstet Gynaecol Res.* 2003;29(1):12–9 10.1046/j.1341-8076.2003.00065.x 12696622

[12] Komisaruk B Carlos BF Beverly W: The Science of Orgasm.JHU Press,2006 For an interview with two of the researchers, see Exploring the Mind-Body Orgasm. Last accessed 26 Nov. 2011. 10.1192/bjp.191.4.369

[13] Mah K Binik YM: Are orgasms in the mind of the body? Psychosocial versus physiological correlates of orgasmic pleasure and satisfaction. *J Sex Marital Ther.* 2005;31(3):187–200 10.1080/00926230590513401 16020138

[14] Utz-Billing I Kentenich H: Female genital mutilation: an injury, physical and mental harm. *J Psychosom Obstet Gynaecol.* 2008;29(4):225–29 Last accessed: 9 December 2011. 10.1080/01674820802547087 19065392

[15] Almroth L Elmusharaf S El Hadi N: Primary infertility after genital mutilation in girlhood in Sudan: a case-control study. *Lancet.* 2005;366(9483):385–91 10.1016/S0140-6736(05)67023-7 16054938

[16] WHO study group on female genital mutilation and obstetric outcome. Banks E Meirik O Farley T: Female genital mutilation and obstetric outcome: WHO collaborative prospective study in six African countries. *Lancet.* 2006;367(9525):1835–41 10.1016/S0140-6736(06)68805-3 16753486

[17] Larsen U Okonofua FE: Female circumcision and obstetric complications. *Int J Gynecol Obstet.* 2002;77(3):255–65 10.1016/S0020-7292(02)00028-0 12065141

[18] Hakim LY: Impact of female genital mutilation on maternal and neonatal outcomes during parturition. *East Afr Med J.* 2001;78(5):255–8 10.4314/eamj.v78i5.9049 12002086

[19] Geneletti S Richardson S Best N: Adjusting for selection bias in retrospective, case-control studies. *Biostatistics.* 2009 Jan;10(1):17–31 10.1093/biostatistics/kxn010 18482997

[20] Elmusharaf S Elhadi N Almroth L: Reliability of self reported form of female genital mutilation and WHO classification: cross sectional study. *BMJ.* 2006;333(7559):124–27 10.1136/bmj.38873.649074.55 16803943 PMC1502195

[21] Richards D: Male Circumcision: Medical or Ritual?3 JLM at 372; Parekh, op cit n 63, at 268; United Nations Centre for Human Rights, Harmful Traditional Practices Affecting the Health of Women and Children 1996; Fact Sheet No 23 (Geneva, 1995), p. 8;(1996) Reference Source

[22] Dirie MA Lindmark G: The risk of medical complications after female circumcision. *East Afr Med J.* 1992;69(9):479–482 1286628

[23] El Saadawi N: The Hidden Face of Eve: Women in Arab World Women of Arab World,Translated and edited by Sherif Hetata. Published by Zed Books, London,1980;pp. 7–11 Reference Source

