# Supplementary material for: Female circumcision: Limiting the harm
Source: F1000Res. 2012 Nov 8;1:23. Originally published 2012 Oct 5. [Version 2] doi: 10.12688/f1000research.1-23.v2 (PMC3917656; doi:10.12688/f1000research.1-23.v2)
Supplement: Peer review history

# Peer review: Referee response for version 2

**Published:** 2012-11-19

**Authors:** Ali Akoum

**Affiliations:** 1 Department of Obstetrics and Gynecology, Laval University, Québec, PQ, Canada

I personally think that the study is well performed. The author made a fair presentation of the literature and outlined the various studies and statistics on this subject, which, the least we can say is delicate and controversial.

The author presents a point of view that advocates a change in legislation in order to make this practice legal and carried out by professionals in an appropriate and sterile environment, so that to reduce the risk of infection and sequelae. That being said, I think it would have been appropriate to make sense of things and take into account the traditions and cultures of the peoples who perpetuate and believe traditionally and/or religiously in the virtues of this practice and other peoples who do not share this point of view and condemn such practices for equally valid social and cultural reasons.

Perhaps it is better to firstly encourage change, possibly via the WHO, humanitarian organizations, diplomatic channels, etc., and make every effort to ensure that the authorities of the countries concerned put an end to conditions often atrocious, unacceptable and condemnable in which these interventions are made, and the way one treat and mutilate young girls who despite the strong traditions and cultures have fundamental rights that must be respected. If we admit that we cannot change traditions and habits and must avoid to “westernize” the way of life of other peoples and if genital cutting in girls is “mandatory” by the force of tradition and culture in some countries, one must ensure that such interventions are carried out in an environment that allows these girls not to suffer and experience significant physical and psychological distresses. On the other hand, we do not have as a society to change our laws and go against our values to legalize or allow such practice to any group of our fellow citizens, especially as there is no scientific evidence that it improves in anything the well‐being of women and brings an advantage that they cannot do without. Rather, we should ensure that our laws protecting human rights and the physical, moral and psychological integrity of a person are respected.

I have read this submission. I believe that I have an appropriate level of expertise to confirm that it is of an acceptable scientific standard, however I have significant reservations, as outlined above.

# Peer review: Referee response for version 1

**Published:** 2012-10-16

**Authors:** Hisham Kandil

**Affiliations:** 1 Department of Obstetrics and Gynecology, Cairo University, Cairi, Egypt

I approve the validity of this opinion article with some minor remarks.

I personally do not approve of female genital cutting as a general routine, however it is commonly practiced in rural areas of third world countries. Due to this, it is important to study how to best deal with the problem rather than totally deny it. The first degree procedure may be a first step towards avoiding further damage.

I think that the final section ‘final remarks’ should be replaced with the title ‘conclusions’.

I have read this submission. I believe that I have an appropriate level of expertise to confirm that it is of an acceptable scientific standard.

# Peer review: Referee response for version 1

**Published:** 2012-10-11

**Authors:** Ahmed Fetouh

**Affiliations:** 1 Faculty of Medicine, Al-Azhar University for Girls, Cairo, Egypt

My own personal stand is against female genital cutting except as a plastic surgery procedure for restricted indications.

I have read this submission. I believe that I have an appropriate level of expertise to confirm that it is of an acceptable scientific standard.
